# If horses had toes: demonstrating mirror self recognition at group level in *Equus caballus*

**DOI:** 10.1007/s10071-021-01502-7

**Published:** 2021-03-13

**Authors:** Paolo Baragli, Chiara Scopa, Veronica Maglieri, Elisabetta Palagi

**Affiliations:** 1 Department of Veterinary Sciences, University of Pisa, Viale delle Piagge 2, 56124 Pisa, Italy; 2 Research Center “E. Piaggio”, University of Pisa, Largo Lucio Lazzarino 1, 56122 Pisa, Italy; 3 Italian National Reference Centre for Animal Assisted Interventions, Istituto Zooprofilattico Sperimentale delle Venezie, Viale dell’Università 10, 35020 Legnaro, Padua Italy; 4 Unit of Ethology, Department of Biology, University of Pisa, Via Alessandro Volta 4bis, 56126 Pisa, Italy; 5 Natural History Museum, University of Pisa, Via Roma, 79, 56011 Calci, Pisa Italy

**Keywords:** Mark test, Colored mark, Sham mark, Image of self

## Abstract

Mirror self-recognition (MSR), investigated in primates and recently in non-primate species, is considered a measure of self-awareness. Nowadays, the only reliable test for investigating MSR potential skills consists in the untrained response to a visual body mark detected using a reflective surface. Here, we report the first evidence of MSR at group level in horses, by facing the weaknesses of methodology present in a previous pilot study. Fourteen horses were used in a 4-phases mirror test (covered mirror, open mirror, invisible mark, visible colored mark). After engaging in a series of contingency behaviors (looking behind the mirror, peek-a-boo, head and tongue movements), our horses used the mirror surface to guide their movements towards their colored cheeks, thus showing that they can recognize themselves in a mirror. The analysis at the group level, which ‘marks’ a turning point in the analytical technique of MSR exploration in non-primate species, showed that horses spent a longer time in scratching their faces when marked with the visible mark compared to the non-visible mark. This finding indicates that horses did not see the non-visible mark and that they did not touch their own face guided by the tactile sensation, suggesting the presence of MSR in horses. Although a heated debate on the binary *versus* gradualist model in the MSR interpretation exists, recent empirical pieces of evidence, including ours, indicate that MSR is not an all-or-nothing phenomenon that appeared once in phylogeny and that a convergent evolution mechanism can be at the basis of its presence in phylogenetically distant taxa.

**Supplementary Information:**

The online version contains supplementary material available at 10.1007/s10071-021-01502-7.

## Introduction

Since the 1960′s mirror self-recognition (MSR) has been introduced as a measure of the “awareness of self” in great apes and humans (Gallup 1968, 1977a). The methodology is based on a test in which subjects shift from other-directed behaviors towards self-directed behaviors after a certain amount of time of exposure to a mirror. Other-directed behaviors are elicited by the perception of the presence of a conspecific, while self-directed behaviors involve the investigation of body parts normally not visible without the aid of a reflective surface. In these terms, this shift has been interpreted as a mirror-induced demonstration of self-recognition ability (Suárez and Gallup 1981). The only reliable data informing us of the presence of MSR is the untrained response to a visual body mark detected with the assistance of a reflective surface (de Waal 2019). The first successful experiments showing the presence of the phenomenon in non-human primates were obtained on *Pan* and *Pongo* genera (for an extensive review Anderson and Gallup 2015). Within the primate order, the experiments conducted on monkeys revealed no presence of MSR (Gallup 1977a, b), although some recent studies challenge these first results (Rajala et al. 2010; Chang et al. 2017).

Starting from 2000 the studies on MSR have expanded to many vertebrate taxa beside primates, although the application of the classic MSR experimental paradigm has introduced important methodological variants according to the different species tested (de Waal 2019). In bottlenose dolphins (*Tursiops truncatus*) the authors considered as mark-directed behaviors those movements in which the animal positioned itself towards the reflective surface to visually explore the mark (Reiss and Marino 2001). While in other studies touching/scraping the mark has been considered the self-directed behavior proving self-recognition (elephants, *Elephas maximus*, Plotnik et al. 2006; magpies, *Pica pica*, Prior et al. 2008). In cleaner wrasses (*Labroides dimidiatus*), Kohda and coworkers (2019) considered rubbing marked-throat against the substrate as a reliable measure of self-directed behaviors. In their pilot study on the domestic horse (*Equus caballus*), Baragli et al. (2017) considered face scraping and rubbing against the substrate as indicators of mark-directed behaviors.

Overall, the tests on all these species seem to indicate the presence of MSR ability in at least a few subjects, although the issue is still under debate (for extensive review see de Waal 2019; Gallup and Anderson 2019). One of the most criticized issues is the methodological procedure adopted for the demonstration of MSR. Some weaknesses in the experimental protocols have been highlighted by de Waal (2019) and Gallup and Anderson (2019). According to these authors, the studies exploring MSR in non-primate species suffer some methodological biases such as very reduced sample size, not always mirror naive animals, asymmetric arenas, presence of conspecifics during the test, irritating marks, non-blind video analyses and lack of standardization in the number of trials proposed to the different subjects for each step (e.g. subjects that showed a higher level of response were tested more than others). A further critical point of the studies is the almost total absence of their replication on non-primate species, except for bottlenose dolphins (two studies focusing on two adults, (Reiss and Marino, 2001), and two juveniles, Morrison and Reiss 2018). Moreover, analyses at group level are completely missing. The only species tested at group level is the giant panda (*Ailuropoda melanoleuca*) that did not show any evidence of MSR (Ma et al. 2015).

Because of the methodological criticism, we replicated the MSR test in a group of 14 horses by trying to limit the biases present in the pilot study (Baragli et al. 2017; *n* = 4) and by taking into account the weaknesses raised by the most recent literature as much as possible. In the present study, the horses were tested in a symmetric arena without the presence of conspecifics and the number of trials was standardized (one trial *per* step). Moreover, since the pilot study revealed that the motivation to react to the mirror dropped after about 20 min (each trial lasted 60 min in Baragli et al. 2017), in the present study we set each trial at 30 min. None of the tested subjects underwent previous training.

The studied horses were subjected to a 4-phases mirror test. The first two phases (Covered Mirror CM; Open Mirror, OM) were related to the reaction to mirror exposure. In CM and OM, behaviors related to the understanding of mirror functionality were recorded such as selective attention, exploration towards the mirror, and contingency behaviors.

In our approach, we followed the criteria suggested by de Waal (2019) about MSR prerequisites. We went on with the mark test when social reactions to the mirror were replaced by contingency behaviors and we ascertained that self-touching was driven by the visual component of the mark. Horses were marked with an invisible mark positioned on both cheeks (Online Resource 1), an area of the body only visible with the aid of the mirror (third phase, sham condition). In the fourth phase a visible mark (mark condition) was positioned in the same anatomical region (Online Resource 1). In the third and fourth phase, the behavior directed towards the mark (invisible or visible) has been collected (face scratching and rubbing). As a further control, the same behavior directed towards the whole body (excluding the face) has been measured in both sham and mark phases. For the definition of the behaviors collected during the four phases see Table 1.

**Table 1:** Operational definition of the behavioral patterns

Behavior	Definition	Supplementary information	Cohen’s *K* values
*Behaviors towards the mirror*			
Selective attention	The horse maintains its head perpendicular to the mirror surface and its ears directed towards the mirror	Online Resource 2	*K* = 0.98
Exploring mirror	The behavior includes sniffing, licking, biting, touching the mirror using the mouth and the nose as previously defined (Baragli et al. 2017)	Online Resource 3	*K* = 0.94
*Contingency behaviors*			
Looking behind	The horse is close to the mirror (< 1 m) and put its head and neck beyond the fence. The horse turns its head toward the rear side of the mirror as previously defined (Baragli et al. 2017)	Online Resource 4	*K* = 0.90
Peek-a-boo	The horse moves its head out and back in sight of the mirror	Online Resource 5	*K* = 0.87
Head movements	The horse performs a series of vertical and lateral quick movements with the head while looking at the mirror with or without stretching its neck	Online Resource 6	*K* = 1.00
Tongue protrusion	The horse protrudes the tip or a large part of its tongue out of the mouth, without showing teeth	Online Resource 7	*K* = 1.00
*Self-directed behaviors*			
Face scratching (Face-SCR)	The horse rubs its face by using both its ipsi- or contralateral forelimbs or any kind of support (wooden poles, the frame of the mirror, ground)	Online Resource 8, 9, 10, 11, 12	*K* = 1.00
Body scratching (Body-SCR)	The horse rubs any part of its body excluding the face, by using both its ipsi- or contralateral forelimbs or by using any kind of support (wooden poles, the fence, the frame of the mirror)	Online Resource 8, 13, 14	*K* = 1.00

First column: names of the behavioral patterns (and acronyms) performed by the horses and considered in the study. Second column: operational definition of each behavioral pattern. Third column: supporting material illustrating the single pattern. Fourth column: Cohen’s *K* values obtained for each behavioral pattern indicating the degree of agreement between the two observers

## Materials and methods

### Animals and housing

Giving that housing and previous negative interactions with humans seem to have an impact on the psychological development of horses (Fureix et al. 2012; Baragli et al. 2014), the subjects were selected based on conventional training procedures and appropriate housing.

The experimental design was conducted in April 2017 at the “Pelliccia” Riding Centre (San Marcello Pistoiese, Tuscany, Italy). The tested animals (14 horses of different ages and breeds, see Online Resource 15) were selected based on features already defined in the pilot study. The horse selection was made on the basis of their propensity to familiarity towards people and confidence with the arena in which the test would be performed. Moreover, the predisposition to explore unfamiliar objects was taken into account (see Baragli et al. 2017 for details). The horses were stabled in individual stalls and had paddock turnout every day in a social environment; they showed no stereotyped behaviors and had the same feeding schedule (ad libitum access to hay and water, grass during paddock turnout and concentrates one time a day).

### Ethic statements

This study was carried out in accordance with the recommendations of the Italian Animal Care Act (Decree Law 26/2014). The Ethical Committee on Animal Experimentation of the University of Pisa approved the experimental protocol (ref. n. 62,131). The owners gave written consent to the use of their horses in this experiment.

### The testing area

The entire experimental design has been performed in a covered arena (the “round pen”, typical circular enclosure usually employed for the training of horses). The arena has been divided into two parts: the testing area and an out-of-testing area (behind the mirror and lateral areas).

**Fig. 1:**
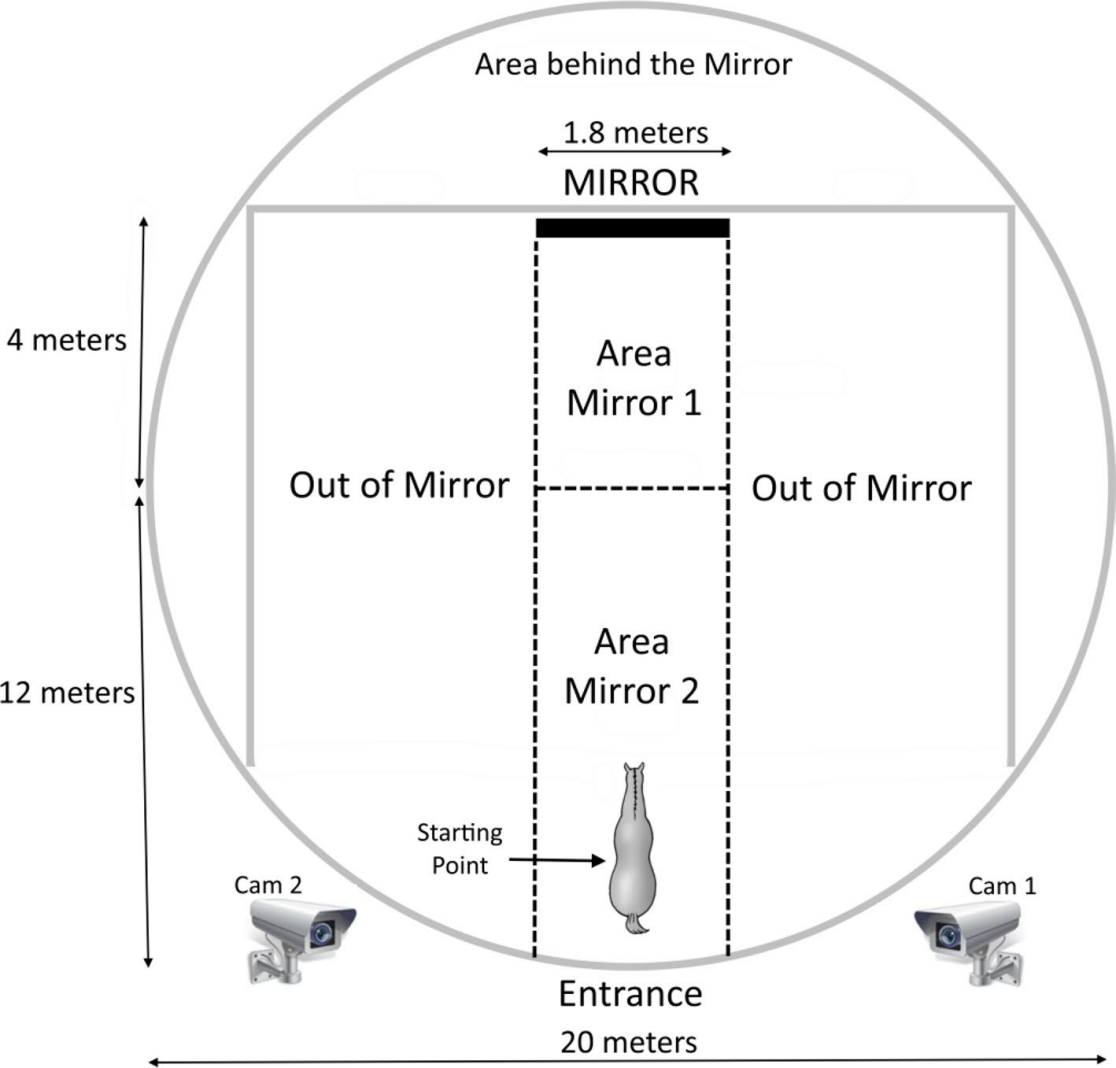
The covered area where all the experimental trials have been conducted. In the figure the four sectors of the area are indicated

The testing area has been further divided into four areas. These four areas have been defined depending on the relative position respect to the mirror (Fig. 1). The starting position was in line with the mirror and the space on the right and on the left from this line was symmetric. This allowed us avoiding environmental lateral biases.

### Experimental design

We tested each horse individually. The tested horse was led to the starting point by the caretaker and it was let free after halter removal. The experimental design comprised four phases preceded by a familiarization period. In this period, the arena was set as in the experimental phases without the presence of the mirror. Although all horses were accustomed to the covered arena, we started with this familiarization period to exclude the presence of undesirable behaviors (frustration and stress-related behaviors).

The four experimental phases:

- Phase 1 (Covered Mirror, CM; day 1). In this phase, the mirror was positioned in the location in which it remained for the whole duration of the study with the reflective surface facing outwards.
- Phase 2 (Open Mirror, OM; day 2). The reflective surface of the mirror was turned towards the testing area thus facing the mirror area 1 and 2 (Fig. 1). Therefore, the tested horse could perceive its image in the mirror.
- Phase 3 (Sham, S, day 3). A transparent cross-shaped  figure (10 cm) was applied on both cheeks of the tested horse (Online Resource 1). The figure consisted of ultrasound water gel (Ultrasound gel, Gima, Milan, Italy). This was necessary to exclude the possibility that the animal's behavior was caused by the tactile or olfactory sensation of the mark rather than the visual mark itself.
- Phase 4 (Mark, M; day 4). The cross-shaped figure on both cheeks were colored by adding a small quantity of yellow or blue odorless, hypoallergenic finger paint (F.I.L.A.—Fabbrica Italiana Lapis ed Affini S.p.A., Milan, Italy) to the transparent ultrasound water gel (Ultrasound gel, Gima, Milan) (Online Resource 1). The selection of two primary colors (yellow or blue) to mark the cheeks of the horse was based on horse color perception (Blackmore et al. 2008). To maximize the chromatic contrast and increase the probability that the subject could actually perceive the colored mark as different from the transparent one, we selected blue or yellow eye-shadow powder in relation to coat color (Baragli et al. 2017).

Between two consecutive tests, the mirror surface was cleaned using a hypoallergenic, odorless detergent to limit the body odors of the animal previously tested. Feces were removed at the end of each test.

Each horse was tested at the same time on consecutive days. Each phase lasted 30 min and began when the halter was removed from the tested horse in the starting position.

The marks (both sham and colored) were placed on both cheeks because the panoramic visual field of horses does not cover this head area (Saslow 1999) and, therefore, the mark could be seen by the tested horse only with the guidance of the mirror. The choice to arrange the mark on the cheek also relied on the easiness for the horse to reach that area by the limbs or by the use of environmental supports.

To standardize the marking procedure (size, shape and tactile sensation), we used three identical cross-shaped foam rubber stamps (sham, blue and yellow, 10 × 10 cms, Online Resource 1).

Before each phase, a 10-min grooming session was performed on the whole body to exclude the possibility that the horse felt that it was marked in a specific area (Anderson and Gallup 2015). Fifteen minutes before the SHAM and MARK phases, the caretaker applied the mark (sham, yellow or blue). Concurrently, a repellent substance (Tri Tec, Chifa srl, Angera, VA) was applied on the whole body of the horse to avoid insect disturbance.

During the test, nobody was present in the testing area. Immediately after the release of the horse caretakers moved into the service room where they had the possibility to control the progress of the test by remote control cameras.

### Data collection and analysis

From the videos collected in the Covered Mirror and Open Mirror conditions the duration of the selective attention, exploration and contingency behaviors (head movements, look behind, peek-a-boo and tongue protrusion) were extracted. While in the Sham and Mark conditions, the duration of scratching the face (Face-SCR) and the body (Body-SCR) was recorded. The behaviors analyzed and their definitions are reported in Table 1.

The videos collected during each trial were analyzed by one of the authors (C.S.). To check for inter-observer agreement and reliability over scoring, 24 randomly selected 5-min segments of videotapes were assigned to another observer, expert in horse behavior and unaware of the aim of the study (Cohen's kappa was never below 0.87 for each behavioral pattern defined in the ethogram, Table 1).

Via Kinovea (0.8.15 version) and VLC (3.0.6 version) with the plugin Jump-to-Time extension, we analyzed the 22 h of videos collected during the four conditions for each of the tested subjects.

Depending on the data distribution parametric (Kolmogorov–Smirnov test *p* > 0.05; Paired Sample t test) or non-parametric (Kolmogorov–Smirnov test *p* < 0.05; Wilcoxon Signed Rank test) tests were applied for the analysis at a group level. For the analysis at an individual level Chi-Square “Goodness of Fit” test (expected frequencies higher than 5.0) was used. Statistical analyses were performed via SPSS (20.0) and VassarStats website (http://vassarstats.net/).

## Results

Three out of the 14 horses did not shift from social response to contingency behaviors in the presence of the reflective surface. In presence of the mirror, one horse showed a strong fearful reaction and remained in the farthest spot of the arena; while the other two reacted in a very aggressive way. According to de Waal (2019), these subjects were excluded from the analyses. To test the duration of each behavior collected at the individual level, we applied the Chi-Square “Goodness of Fit” test (expected frequencies > 5.0). To check for the differences in duration at group level, paired sample *t*-test and Wilcoxon signed-rank test were employed according to the data distribution.

## Covered mirror (CM) vs open mirror condition (OM)

### Analysis at an individual level

*Selective attention.* Ten out of eleven horses payed a higher amount of selective attention towards the mirror in OM than in the CM condition (Table 2).

**Table 2:** Results of the of Selective attention and exploration at individual level

Horses	Selective attention			Exploration		
	CM	OM	Χ^2^; *p*	CM	OM	Χ^2^; *p*
Antonia	2.0	271.6	Χ^2^ = 263.7; *p *< .0001	4.3	263.0	Χ^2^ = 248.4; *p* < .0001
Arramon	9.3	175.5	Χ^2^ = 147.7; *p* < .0001	21.6	339.5	Χ^2^ = 278.1; *p* < .0001
Ercole	395.2	345.3	Χ^2^ = 3.2; *p* = 0.072	10.6	584.4	Χ^2^ = 551.4; *p* < .0001
Falco2	555.3	1068.7	Χ^2^ = 161.7; *p* < .0001	0.0	0.0	N/A
King	59.0	110.8	Χ^2^ = 15.2; *p* < .0001	11.0	438.0	Χ^2^ = 314.5; *p* < .0001
Naidjia	184.7	436.8	Χ^2^ = 101.5; *p* < .0001	36.6	67.2	Χ^2^ = 8.44; *p* = 0.004
Oliver	144.1	481.4	Χ^2^ = 180.2; *p* < .0001	0.0	571.3	Χ^2^ = 569.3; *p* < .0001
Oti	56.9	288.5	Χ^2^ = 154.0; p < .0001	35.8	436.4	Χ^2^ = 338.2; p < .0001
Serafine	42.1	425.0	Χ^2^ = 312.2; *p* < .0001	26.9	349.4	Χ^2^ = 274.7; *p* < .0001
Shaif	20.6	745.5	Χ^2^ = 684.0; *p* < .0001	0.0	39.8	Χ^2^ = 37.8; *p* < .0001
Sunshine	42.4	270.8	Χ^2^ = 165.1; *p* < .0001	125.5	0.0	Χ^2^ = 123.5; *p* < .0001

Results of the individual analyses (Chi-Square, Χ^2^;* p* values) of Selective Attention and Exploration in Covered Mirror (CM) and Open Mirror conditions (OM). Values in CM and OM columns are reported in seconds. N/A: not shown as having a meaningful interpretation. N/A: not applicable

*Mirror exploration*. Eight out of eleven horses explored the mirror under the CM condition, one of them performed the behavior significantly more in the CM than in the OM condition. Nine out of eleven horses spent a significantly longer time in exploring the mirror in the OM than the CM condition (Table 2).

*Contingency behaviors.* Three out of eleven horses looked behind the mirror under the CM condition, while in the OM condition eight horses performed this behavior. Six out of eight horses looked behind the mirror significantly more in the OM than in the CM condition (Table 3). In the OM condition, nine out of eleven horses performed repetitive head movements, while only three horses did it in the CM condition. Six out of nine horses engaged in head movements for longer in the OM compared to the CM condition (Table 3). The peek-a-boo was performed by nine out of eleven horses in the OM condition, while none of the horses performed peek-a-boo in the CM condition. Four horses reached statistical significance at the individual level when peek-a-boo was compared between the two conditions (Table 3). Tongue protrusion was performed by two horses in the OM while this behavior was never performed in the CM condition. One of the two horses engaged in this behavior more in the OM than in the CM (Table 3).

**Table 3:** Analyses at individual level for the contingency behaviors

Horses	Head movements			Peek-a-Boo			Look behind			Tongue protrusion		
	CM	OM	Χ^2^; *p*	CM	OM	Χ^2^; *p*	CM	OM	Χ^2^; *p*	CM	OM	Χ^2^; *p*
Antonia	0.0	0.0	N/A	0.0	4.9	N/A	0.0	4.6	N/A	0.0	10.1	Χ^2^ = 8.2; *p* = 0.004
Arramon	0.0	2.5	N/A	0.0	2.6	N/A	13.7	29.8	Χ^2^ = 5.2; *p* = 0.0221	0.0	0.0	N/A
Ercole	0.0	25.9	Χ^2^ = 23.9; *p* < .0001	0.0	2.0	N/A	0.0	24.6	Χ^2^ = 22.6; *p* < .0001	0.0	0.0	N/A
Falco2	15.6	31.1	Χ^2^ = 4.5; *p* = 0.034	0.0	3.1	N/A	0.0	0.0	N/A	0.0	0.0	N/A
King	3.7	1.9	N/A	0.0	27.9	Χ^2^ = 25.9; *p* < .0001	0.0	20.8	Χ^2^ = 18.8; *p* < .0001	0.0	0.0	N/A
Naidjia	0.0	29.7	Χ^2^ = 27.7; *p* < .0001	0.0	0.0	N/A	2.2	23.5	Χ^2^ = 16.0; *p* < .0001	0.0	0.0	N/A
Oliver	0.0	6.7	N/A	0.0	18.6	Χ^2^ = 16.7; *p* < .0001	0.0	4.2	N/A	0.0	0.0	N/A
Oti	0.0	0.0	N/A	0.0	2.2	N/A	0.0	22.2	Χ^2^ = 20.2; *p* < .0001	0.0	0.0	N/A
Serafine	0.0	15.6	Χ^2^ = 13.7; *p* < .0001	0.0	18.6	Χ^2^ = 16.7; *p* < .0001	2.9	8.1	Χ^2^ = 1.6; *p* = 0.206	0.0	0.4	N/A
Shaif	2.2	26.4	Χ^2^ = 18.8; *p* < .0001	0.0	30.2	Χ^2^ = 28.2; *p* < .0001	0.0	0.0	N/A	0.0	0.0	N/A

Results of the analyses (Chi-Square, Χ^2^; *p* values) at individual level for the four contingency behaviors recorded in the Covered Mirror (CM) and Open Mirror (OM) conditions. Values in CM and OM columns are reported in seconds. N/A: not applicable

### Analysis at a group level

To understand if the reflective surface determined a variation in the selective attention, mirror exploration, and contingency behaviors (look behind, head movements, peek-a-boo and tongue protrusion), we compared the time spent (in seconds) in these activities between the CM and the OM condition at a group level.

*Selective attention.* Horses were longer attentive towards the mirror in the OM (mean 420.0 ± 82.8 SE) than in the CM condition (mean 135.6 ± 54.2 SE), thus suggesting that the reflective surface has a role in prolonging the duration of this behavior (*t* =  – 4.454; df = 10; *p* = 0.001).

*Mirror exploration*. Under the CM condition, the time spent in the exploratory activity towards the mirror was much less (mean 24.8 ± 10.9 SE) than in the OM condition (mean 280.8 ± 67.1 SE). Thus, the presence of the reflective surface induced an increase in the exploration of the mirror (*t* =  – 3.565, df = 10, *p* = 0.005).

*Contingency behaviors.* Horses looked behind the mirror for longer in the OM (mean 13.3 ± 3.9 SE) than in the CM (mean 1.7 ± 1.2 SE) condition (*t *=   –  3.548; df = 10; *p* = 0.005). The head movements lasted significantly longer in the OM (mean 16.2 ± 4.4 SE) than in the CM (mean 2.0 ± 1.4 SE) condition (*t* =  – 3.413, df = 10, *p* = 0.007). We found the same for peek-a-boo that lasted longer during the OM (mean 10.0 ± 3.5 SE) than the CM (mean 0.0 ± 0.0 SE) condition (*t* =  – 2.879, df = 10, *p* = 0.016). At a group level tongue protrusion did not reveal any statistical difference between the CM (mean 0.0 ± 0.0 SE) and the OM (mean 1.0 ± 0.9 SE) condition (Z =  – 1.342; ties = 9; *p* = 0.180).

## Sham (S) vs Mark (M) condition

### Analysis at an individual level

*Face Scratching* (Face-SCR). Nine out of eleven horses scratched their face in the M condition; while, in the S condition, five out of eleven horses scratched their face. Three out of four tested horses spent a significantly higher amount of time scratching their face in M compared to S condition (Table 4).

**Table 4:** Analyses at individual level of face and body scratching

Horses	Face-SCR			Body-SCR		
	Sham	Mark	Chi-square, *p*-values	Sham	Mark	Chi-square, *p*-values
Antonia	0.0	7.5	N/A	0.0	1.4	N/A
Arramon	0.0	0.0	N/A	4.5	5.0	N/A
Ercole	3.7	15.6	Χ^2^ = 6.2; *p* = 0.013	17.1	20.3	Χ^2^ = 0.1; *p* = 0.729
Falco2	3.7	7.0	Χ^2^ = 0.5; *p* = 0.480	0.0	0.0	N/A
King	0.0	5.0	N/A	3.7	0.0	N/A
Nadijia	0.0	4.3	N/A	2.7	3.6	N/A
Oliver	0.0	4.8	N/A	6.6	7.1	Χ^2^ = 0.0; *p* = 1.000
Oti	0.0	5.1	N/A	21.3	5.5	Χ^2^ = 8.2; *p* = 0.004
Serafine	4.0	0.0	N/A	0.0	5.0	N/A
Shaif	1.1	9.0	Χ^2^ = 4.7; *p *= 0.030	30.3	7.2	Χ^2^ = 13; *p* < 0.001
Sunshine	1.7	10.1	Χ^2^ = 4.6; *p* = 0.031	8.2	4.5	Χ^2^ = 0.6; *p* = 0.446

Results of the analyses carried out at individual level of face (Face-SCR) and body (Body-SCR) scratching in the Sham and Mark conditions. For the Face-SCR duration (in seconds), the significant results indicate a difference in favor of the Mark compared to the Sham condition (Face-SCR Mark > Face-SCR Sham). For the Body-SCR duration (in seconds), the significant results indicate a difference in favor of the SHAM compared to the MARK condition (Body-SCR Sham > Body-SCR Mark).

*Body Scratching* (Body-SCR)***. ***Nine out of eleven horses scratched their body in the M condition; while, in the S condition, eight out of eleven horses scratched their body. Two out of five horses spent a significantly less amount of time in scratching their body in the M compared to the S condition (Table 4).

### Analysis at a group level

*Face Scratching* (Face-SCR). In the presence of the reflective surface, horses spent significantly more time (in seconds) in scratching their faces in M (visible color mark; mean 6.21 ± 1.34 SE) compared to the S (transparent mark; mean 1.29 ± 0.51 SE) condition (*t *=  – 3.3139, df = 10, *p* = 0.011).

*Body Scratching* (Body-SCR)*.* No significant difference was found comparing the time spent in body scratching between the M (mean 5.42 ± 1.67 SE) and the S (mean 8.58 ± 3.02 SE) condition (*t* = 1.392, df = 10, *p* = 0.194).

## Discussion

Here, we report the first evidence of mirror self-recognition at the group level in a non-primate species. Furthermore, using a larger sample size and applying a more accurate experimental procedure, the present study replicates a previous pilot study on mirror self-recognition in horses (Baragli et al. 2017).

Our horses used the mirror surface to guide their movements towards their faces previously marked, thus showing that they are able to recognize themselves in a mirror. They followed a sequence of behavioral steps towards the mirror before being marked. This is a fundamental criterion to be fulfilled before undergoing the mark test, as suggested by de Waal (2019), Gallup et al. (2002) and Gallup and Anderson (2019) in their reviews focused on the methodological issues. These steps are indicative of the cognitive processes leading animals to understand that the image reflected in the mirror is the image of self (Plotnik et al. 2006).

Firstly, we found that in presence of the reflective surface the behavior of the horses clearly differed when compared to the condition in which the surface was covered. Both selective attention and exploratory activity increased when the mirror was open, indicating the emergence of the violation of the expectancy phenomenon (Seyfarth et al. 2005; Poulin-Dubois et al. 2009; Kondo et al. 2012). Through the violation of expectancy paradigm, it has been demonstrated that horses are able to associate multiple sensory cues to recognize conspecifics and people (cross-modal recognition, Proops et al. 2009; Proops and McComb 2012). While the image in the mirror satisfied the visual criterion (*there is a horse in the mirror* sensu Lorenz 1974), the tactile and olfactory information did not match with the visual one (*it is not a horse* sensu Lorenz 1974) thus producing an incongruent set of information.

The information gathered by the selective attention and exploratory activities increased the horse’s motivation in engaging in contingency behaviors to solve such incongruency (Seyfarth et al. 2005). The so-called contingency behaviors include highly repetitive non-stereotyped or unusual movements only when animals are in front of the reflective surface, probably to verify if the movements of the image in the mirror match their own movements. When in front of the mirror, magpies moved their head or body back and forth (Prior et al. 2008), elephants displayed repetitive, non-stereotypic trunk and body movements (Plotnik et al. 2006), jackdows and crows showed “peek-a boo” movements during which the bird moved out and back in sight of the mirror (Soler et al. 2014; Vanhooland et al. 2019) and chimpanzees manipulated their lips and tongues while glancing into the mirror (Povinelli et al. 1993). Our horses engaged in contingency behaviors similar to those reported for other species such as head movements, peek-a-boo, and tongue protrusion almost exclusively in presence of the reflective surface (Table 3). It is possible that by slightly moving their head horses managed to avoid the blind spot characterizing their frontal view (Lansade et al. 2020) thus head movements could help verify whether the movements of the reflective image corresponds to their movements (Online Resource 6). One of the most indicative contingency behaviors reported in the literature is looking behind the mirror that is enacted to verify the possible presence of a conspecific behind the reflective surface (*Pica pica*, Prior et al. 2008; *Equus caballus*, Baragli et al. 2017; *Loxodonta africana*, Plotnik et al. 2006; *Pan troglodytes*, Gallup 1970; Povinelli et al. 1993) (Online Resource 5). Our horses showed a high inter-individual variability in performing contingency behaviors in front of the reflective surface. We suggest that the strategy employed to test the mirror function varies among subjects that engaged in one or two contingency behaviors to solve the violation of expectancy (Table 3). This means that when studying MSR we should take into account for this variability by also checking a posteriori what animals do to test their own image reflected in the mirror (unusual, repetitive non-stereotyped behaviors), thus leaving open the ethogram fixed a priori.

After solving the violation of expectancy by engaging in contingency behaviors, animals gather the necessary information to potentially pass the mark test. In this study, due to the anatomical features limiting the degree of freedom of horses to reach specific areas of their face, we considered scratching the face (Face-SCR) as an attempt to remove the mark which was placed on both cheeks (bilateral marking) (Online Resource 1 and 9–12). The analysis at a group level showed that horses spent a longer time in scratching their face when marked with the colored mark compared to the sham mark (S vs M conditions). This finding indicates that horses did not see the sham mark and that it was not the tactile sensation that induced the animal to touch its own face. The increased level of Face-SCR during the M condition suggests that by using the reflective surface the animals were able to visually perceive the colored spot on their face. The standardization of the procedure preceding the application of the mark, such as grooming on the whole body and identical shapes of the sham and colored stamps, guarantees that the use of the transparent mark worked as an effective control condition. An additional control in supporting the hypothesis that horses are able to perceive the colored spot on their face resides in the comparable levels of time spent in scratching directed to the rest of the body (Body-SCR). In the M condition, scratching appears to be highly directional towards a specific target: the colored face (Online Resource 16).

One of the novelties of the present study relies on the analysis at a group level, which ‘marks’ a turning point in the analytical technique of MSR exploration. It has been suggested that the individual variability in the MSR tests can reflect the low motivation of animals to remove the colored mark. The low motivation to react to the mark can introduce a strong individual bias in the accurate measurement of self-recognition abilities (Bard et al. 2006; Heschl and Burkart 2006). In our case, for example, four horses that did not scratch their faces in the S condition did it in the M condition but not for sufficient time to apply an individual test (expected frequencies < 5.0 s; see Table 4). The behavioral motivation of removing something from one’s own body, and to respond to the colored mark, is considered a hotspot in the discussion about the validity of the mark test for demonstrating MSR. In this perspective, the analysis at the population level provides the opportunity to employ larger samples also including the subjects showing low levels of motivation. Such individual motivation can also be affected by a series of species-specific features (e.g., anatomical difference in properly reaching the marked area, visual perception of specific colors, visual acuity, predominant sensory modality different from vision), including personality and cognitive style. Therefore, the sensory and cognitive systems, as well as the motivation to behaviorally respond to the mark, are substantial preconditions to keep in mind when we decide to test animals’ self-recognition abilities.

In conclusion, despite the strong inter-individual variability, our results suggest the presence of MSR in horses. Although the heated debate on the binary *versus* gradualist model in the MSR interpretation (de Waal 2019; Gallup and Anderson 2019; Brandl 2016), recent empirical pieces of evidence, including ours on horses, indicate that MSR is not an all-or-nothing phenomenon suddenly emerged in the phylogeny, but it has probably been favored by natural selection to adaptively respond to social and cognitive challenges an animal has to cope with.

## Supplementary Information

Below is the link to the electronic supplementary material.

Supplementary file1 (DOCX 25 KB)

Supplementary file2 (PDF 885 KB)

Supplementary file3 (PDF 545 KB)

Supplementary file4 (MP4 2828 KB)

Supplementary file5 (MP4 5412 KB)

Supplementary file6 (MP4 10860 KB)

Supplementary file7 (MP4 4241 KB)

Supplementary file8 (MP4 3863 KB)

Supplementary file9 (PDF 1203 KB)

Supplementary file10 (MP4 7623 KB)

Supplementary file11 (MP4 2619 KB)

Supplementary file12 (MP4 3000 KB)

Supplementary file13 (MP4 10581 KB)

Supplementary file14 (MP4 2574 KB)

Supplementary file15 (MP4 2391 KB)

Supplementary file16 (PDF 35 KB)

Supplementary file17 (AVI 5942 KB)

Supplementary file18 (XLSX 116 KB)

## Declarations

### Availability of data and material

All relevant data, including raw data (Online Resource 17) and example videos, are located in the Supporting Information. The videos that support the findings of this study are available from the corresponding author upon reasonable request.
