# Correction to: If horses had toes: demonstrating mirror self recognition at group level in *Equus caballus*

**DOI:** 10.1007/s10071-022-01650-4

**Published:** 2022-06-30

**Authors:** Paolo Baragli, Chiara Scopa, Veronica Maglieri, Elisabetta Palagi

**Affiliations:** 1 Department of Veterinary Sciences, University of Pisa, Viale delle Piagge 2, 56124 Pisa, Italy; 2 Research Center “E. Piaggio”, University of Pisa, Largo Lucio Lazzarino 1, 56122 Pisa, Italy; 3 Italian National Reference Centre for Animal Assisted Interventions, Istituto Zooprofilattico Sperimentale delle Venezie, Viale dell’Università 10, 35020 Legnaro, Padua Italy; 4 Unit of Ethology, Department of Biology, University of Pisa, Via Alessandro Volta 4bis, 56126 Pisa, Italy; 5 Natural History Museum, University of Pisa, Via Roma, 79, 56011 Calci, Pisa Italy

## Correction to: Animal Cognition (2021) 24:1099–1108 10.1007/s10071-021-01502-7

In the original publication of the article, the table (Table 3) has been incorrectly published, this has been corrected in this paper (Table 3).

**Table 3:** Analyses at individual level for the contingency behaviors

Horses	Head movements			Peek-a-boo			Look behind			Tongue protrusion		
	CM	OM	*Χ*^2^; *p*	CM	OM	*Χ*^2^; *p*	CM	OM	*Χ*^2^; *p*	CM	OM	*Χ*^2^; *p*
Antonia	0.0	0.0	N/A	0.0	4.9	N/A	0.0	4.6	N/A	0.0	10.1	*Χ*^2^ = 8.2; *p* = 0.004
Arramon	0.0	2.5	N/A	0.0	2.6	N/A	13.7	29.8	*Χ*^2^ = 5.2; *p* = 0.0221	0.0	0.0	N/A
Ercole	0.0	25.9	*Χ*^2^ = 23.9; *p* < 0.0001	0.0	2.0	N/A	0.0	24.6	*Χ*^2^ = 22.6; *p* < 0.0001	0.0	0.0	N/A
Falco2	15.6	31.1	*Χ*^2^ = 4.5; *p* = 0.034	0.0	3.1	N/A	0.0	0.0	N/A	0.0	0.0	N/A
King	3.7	1.9	N/A	0.0	27.9	*Χ*^2^ = 25.9; *p* < 0.0001	0.0	20.8	*Χ*^2^ = 18.8; *p* < 0.0001	0.0	0.0	N/A
Naidjia	0.0	29.7	*Χ*^2^ = 27.7; *p* < 0.0001	0.0	0.0	N/A	2.2	23.5	*Χ*^2^ = 16.0; *p* < 0.0001	0.0	0.0	N/A
Oliver	0.0	6.7	N/A	0.0	18.6	*Χ*^2^ = 16.7; *p* < 0.0001	0.0	4.2	N/A	0.0	0.0	N/A
Oti	0.0	0.0	N/A	0.0	2.2	N/A	0.0	22.2	*Χ*^2^ = 20.2; *p* < 0.0001	0.0	0.0	N/A
Serafine	0.0	15.6	*Χ*^2^ = 13.7; *p* < 0.001	0.0	18.6	*Χ*^2^ = 16.7; *p* < 0.0001	2.9	8.1	*Χ*^2^ = 1.6; *p* = 0.206	0.0	0.4	N/A
Shaif	2.2	26.4	*Χ*^2^ = 18.8; *p* < 0.0001	0.0	30.2	*Χ*^2^ = 28.2; *p* < 0.0001	0.0	0.0	N/A	0.0	0.0	N/A
Sunshine	0.0	38.3	*Χ*^2^ = 36.3; *p* < 0.0001	0.0	0.0	N/A	0.0	0.0	N/A	0.0	0.0	N/A

Results of the analyses (Chi-Square, *Χ*^2^; *p* values) at individual level for the four contingency behaviors recorded in the covered mirror (CM) and open mirror (OM) conditions. Values in CM and OM columns are reported in seconds

*N/A* not shown as having a meaningful interpretation

