# Supplementary material for: If horses had toes: demonstrating mirror self recognition at group level in Equus caballus
Source: Anim Cogn. 2021 Mar 13;24(5):1099–108. doi: 10.1007/s10071-021-01502-7 (PMC8360890; doi:10.1007/s10071-021-01502-7)
Supplement: Supplementary file 1 — Supplementary file1 (DOCX 25 KB) [file 10071_2021_1502_MOESM1_ESM.docx]

ESM_3 (separate file). Exploring Mirror: King explores the mirror surface. The breathe of the horse forms a tarnished spot on the surface, thus making simple to detect the exploratory activity (consisting in sniffing and licking).

ESM_4 (separate file). Looking Behind the mirror: Ercole looks behind the mirror by stretching his head and neck beyond the fence, first on the left and then on the right of the mirror.

ESM_5 (separate file). Peek-a-boo: Shaif performs a peek-a-boo by quickly moving his head outside and inside the mirror frame.

ESM_6 (separate file). Head Movements: Oliver performs rapid head movements (up and down) while selectively looking in the mirror.

ESM_7 (separate file). Tongue Protrusion: Antonia repeatedly moves her tongue in and out the mouth. Chewing or exploratory activity did not occur before this behavior.

ESM_9 (separate file). Face Scratching on limb: Falco rubs his marked face on the forelimb after looking in the mirror. Then, after scratching, he looks again in the mirror.

ESM_10 (separate file). Face Scratching on wooden pole: Shaif rubs his marked face on the wooden pole, on the right side.

ESM_11 (separate file). Face Scratching on the mirror frame: Sunshine rubs her marked left cheek and chin on the frame of the mirror.

ESM_12 (separate file). Face Scratching on the ground: Falco rolls on the ground and simultaneously rubs his marked left cheek on the ground. Then, he looks in the mirror and shakes his head and neck. It is quite visible the mark partially removed on the left cheek after Face Scratching.

ESM_13 (separate file). Body Scratching on hindlimb: Nadijia scratches her left hindlimb using teeth.

ESM_14 (separate file). Body Scratching on forelimb: Sunshine scratches his right forelimb using teeth.

ESM_16 (separate file). Face Scratching: In this clip Ercole rubs his marked face, scratching both cheeks on the forelimb. Before rubbing the face, Ercole looks into the mirror and shakes his head. We cannot exclude the possibility that head shaking, having the function of shaking off something from the body, is an attempt of removing the mark before actively scraping it. The position of the camera behind the mirror allows a direct view perspective on the Face Scratching behavior; moreover, it is quite visible the mark partially removed on the right cheek after Face Scratching.

ESM_17 (separate file). The dataset contains all the necessary information of the collected data, including the name of the video files as stored and the timing of occurrence of the behavior, as well as the duration of the behavior. All the 22 hours of videos collected are available upon reasonable request.
