# Supplementary material for: If horses had toes: demonstrating mirror self recognition at group level in Equus caballus
Source: Anim Cogn. 2021 Mar 13;24(5):1099–108. doi: 10.1007/s10071-021-01502-7 (PMC8360890; doi:10.1007/s10071-021-01502-7)
Supplement: Supplementary file 2 — Supplementary file2 (PDF 885 KB) [file 10071_2021_1502_MOESM2_ESM.pdf]

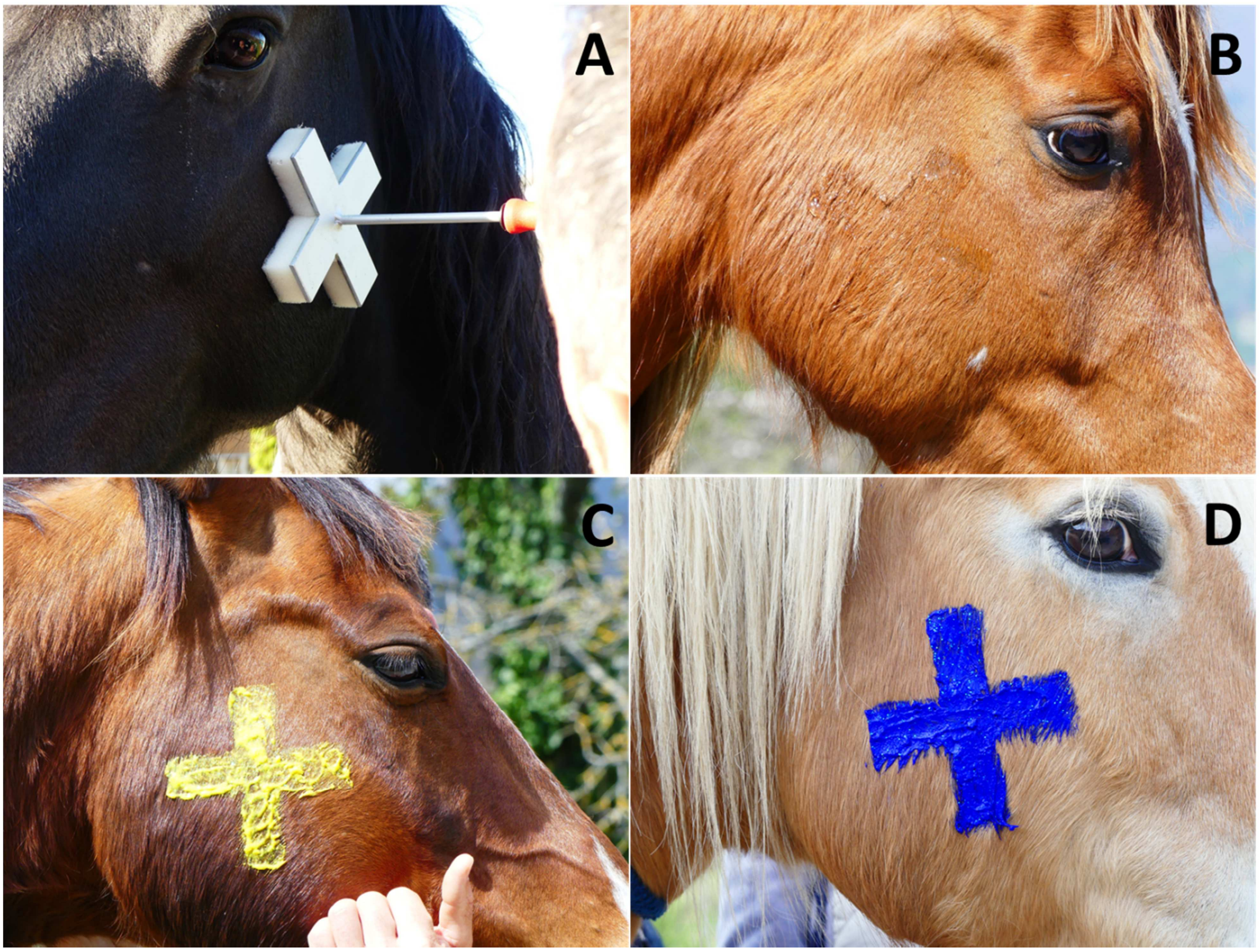

Online Resource 1. (A) procedure used to mark the horses' face, (B) sham mark, (C) yellow marked face, (D) blue marked face.
