# Supplementary figures and images for: If horses had toes: demonstrating mirror self recognition at group level in Equus caballus
Source: Anim Cogn. 2021 Mar 13;24(5):1099–108. doi: 10.1007/s10071-021-01502-7 (PMC8360890; doi:10.1007/s10071-021-01502-7)

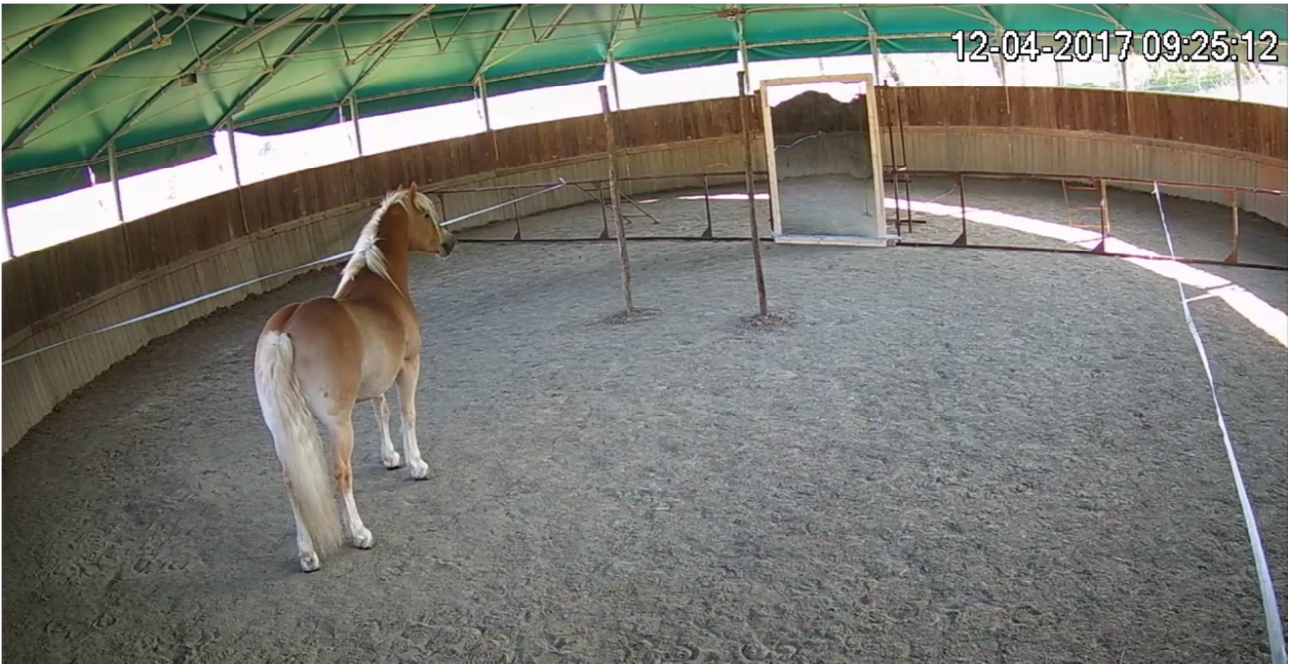

**Online Resource 2.** Oliver performs Selective Attention towards the mirror.

Supplement: Supplementary file 3 — Supplementary file3 (PDF 545 KB) [file 10071_2021_1502_MOESM3_ESM.pdf]
