# Supplementary material for: If horses had toes: demonstrating mirror self recognition at group level in Equus caballus
Source: Anim Cogn. 2021 Mar 13;24(5):1099–108. doi: 10.1007/s10071-021-01502-7 (PMC8360890; doi:10.1007/s10071-021-01502-7)
Supplement: Supplementary file 9 — Supplementary file9 (PDF 1203 KB) [file 10071_2021_1502_MOESM9_ESM.pdf]

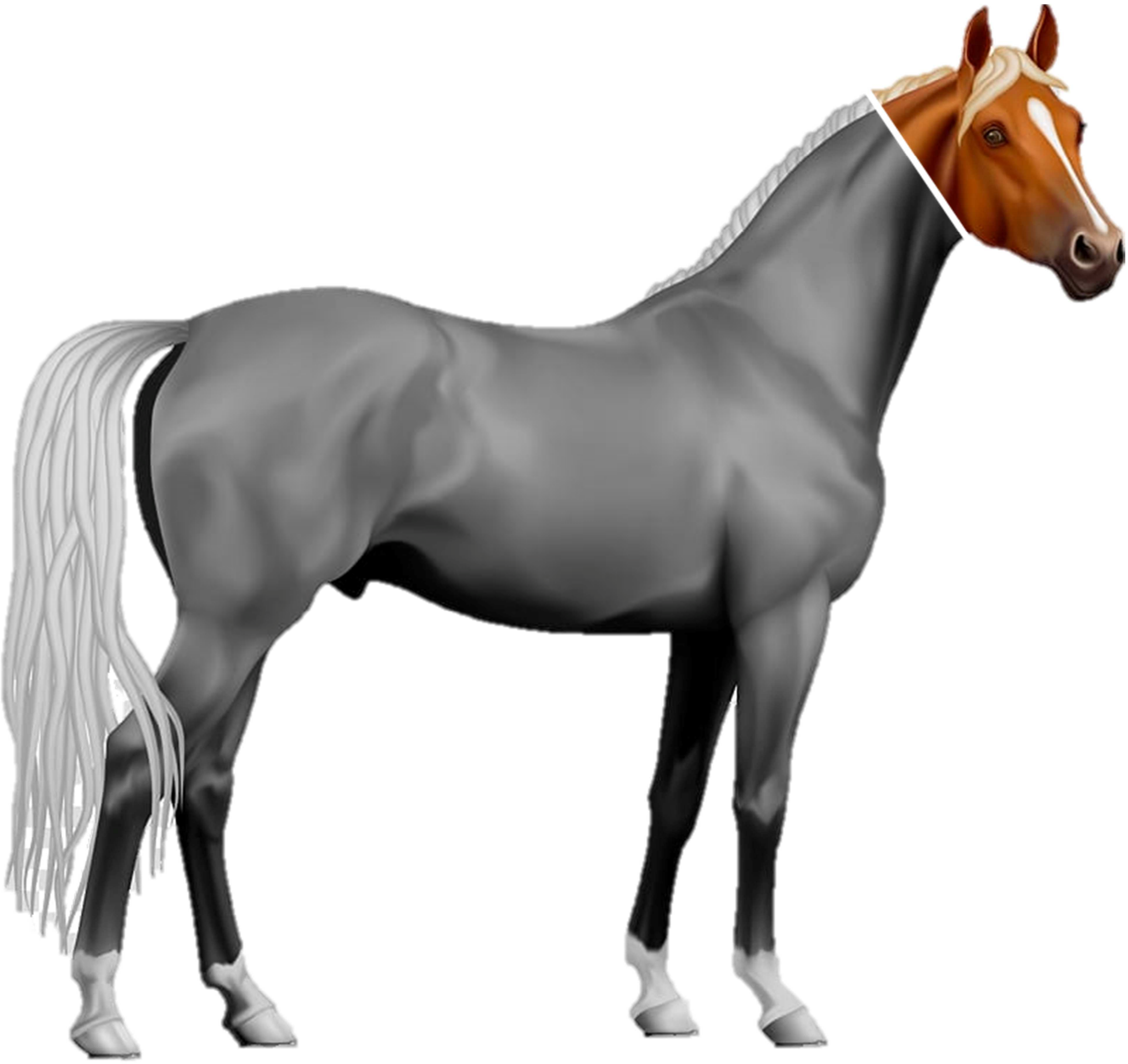

Online Resource 8. Picture showing the Body Scratching area (gray) and Face Scratching area (colored).
