# Supplementary material for: If horses had toes: demonstrating mirror self recognition at group level in Equus caballus
Source: Anim Cogn. 2021 Mar 13;24(5):1099–108. doi: 10.1007/s10071-021-01502-7 (PMC8360890; doi:10.1007/s10071-021-01502-7)
Supplement: Supplementary file 16 — Supplementary file16 (PDF 35 KB) [file 10071_2021_1502_MOESM16_ESM.pdf]

Online Resource 15. List of the tested horses

**Subject**	**Sex**	**Age**	**Breed**
Antonia	Female	8	Spanish Pure Breed
Arramon	Gelding	15	Haflinger
Ercole	Gelding	10	Frisone
Falco2	Gelding	13	Mix breed
King	Gelding	14	Quarter horse
Nadijia	Female	8	Arab horse
Oliver	Gelding	9	Haflinger
Oti	Gelding	13	Haflinger
Serafine	Female	9	Mix breed
Shaif	Gelding	8	Arab horse
Sunshine	Gelding	10	Arab horse
Falco	Gelding	10	Maremmano
Tempesta	Female	4	Mix breed
Dafne	Female	2	Haflinger
